# Survival and predictors of asphyxia among neonates admitted in neonatal intensive care units of public hospitals of Addis Ababa, Ethiopia, 2021: a retrospective follow-up study

**DOI:** 10.1186/s12887-022-03238-w

**Published:** 2022-05-10

**Authors:** Fekadeselassie Belege Getaneh, Girum sebsbie, Mekonen Adimasu, Natnael Moges Misganaw, Desalegn Abebaw Jember, Dires Birhanu Mihretie, Shiferaw Abeway, Zebenay Workneh Bitew

**Affiliations:** 1 College of Health Sciences, Addis Ababa University, Addis Ababa, Ethiopia; 2 College of Health Sciences, Debre Tabor University, Debre Tabor, Ethiopia; 3 St. Paul’s Hospital Millennium Medical College, Addis Ababa, Ethiopia; 4 Dilla University, College of Health Sciences, Dilla, Ethiopia; 5 College of Medicine and Health Sciences, Wollo University, Dessie, Ethiopia

**Keywords:** Perinatal-asphyxia, Predictors, Survival-status, Median recovery time

## Abstract

**Background:**

Globally, perinatal asphyxia (PNA) is a significant cause of most neonatal deaths. Similarly, the burden of birth asphyxia in Ethiopia remains high (22.52%) and has been noted the second leading cause of neonatal mortality. Thus, researches on survival status and predictors of perinatal asphyxia are critical to tackle it. Therefore, the current study intended to determine the survival status and predictors of asphyxia among neonates admitted in Neonatal Intensive Care Units of public hospitals, Addis Ababa, Ethiopia.

**Methods:**

Hospital-based retrospective follow-up study was conducted in four selected public hospitals of Addis Ababa from January 2016 to December 2020. Data were collected using a pretested structured questionnaire. Epi-data 4.6 and STATA Version 16 was used for data entry and analysis, respectively. Kaplan–Meier survival curve, log-rank test and Median time were computed. To find the predictors of time to recovery, a multivariable Cox proportional hazards regression model was fitted, and variables with a P-value less than 0.05 were considered statistically significant. Finally, the Schoenfeld residual test was used to check overall model fitness.

**Result:**

Four hundred eleven admitted asphyxiated babies were followed a total of 3062 neonate-days with a minimum of 1 h to a maximum of 28 days. The Overall incidence density rate of survival was 10 (95% CI: 0.08–0.11) per 100 neonate-days of observation with a median recovery time of 8 days (95% CI: 7.527–8.473). Low birth weight (Adjusted hazard ratio [AHR]: 0.67, 95% CI: 0.47–0.96), stage II hypoxic ischemic encephalopathy (HIE) (AHR: 0.70, 95% CI: 0.51–0.97), stage III HIE (AHR: 0.44, 95% CI: 0.27–0.71), seizure (AHR: 0.61, 95% CI: 0.38—0.97), thrombocytopenia (AHR: 0.44, 95% CI: 0.24–0.80) and calcium gluconate (AHR: 0.75, 95% CI: 0.58–0.99) were found to be independent predictors of time to recovery of asphyxiated neonates.

**Conclusion:**

In the current findings, the recovery time was prolonged compared to others finding. This implies early prevention, strict monitoring and taking appropriate measures timely is mandatory before babies transferred into highest stage of HIE and managing complications are recommended to hasten recovery time and increase survival of neonates.

**Supplementary Information:**

The online version contains supplementary material available at 10.1186/s12887-022-03238-w.

## Introduction

Perinatal Asphyxia is an injury that occurs during the perinatal period due to lack of oxygen flow to the fetus, which may lead to ischemia of the brain or other organs [1]. It can be also defined by using clinical parameters (bag-and-mask ventilation (BMV) at birth or APGAR score ≤ 6) and biochemical parameters (hypercarbia, hypoxemia, and metabolic acidosis (base deficit > 12 mmol/L) due to diminished gas exchange) and end-organ damage [2].

Globally, perinatal-asphyxia is a major cause of morbidity and mortality among newborns. Approximately four million neonates become seriously deprived of oxygen during birth each year [3] and nearly 20 per 1000 deliveries usually require resuscitation, with biochemical and clinical evidence of perinatal asphyxia [4]. The proportion of asphyxiation at birth is ten times greater in developing countries than in developed countries [5]. About 3.6 million (3%) of all infants have moderate to severe PNA at birth, and it contributed to 23% of death among neonates in the developing world according to WHO estimates [6].

Despite the fact that global infant mortality has been progressively reducing for the past two decades, progress in Sub-Saharan Africa has been slow. Perinatal asphyxia is a major cause of newborn deaths in the Sub-Saharan Africa region and now contributes 280,000 deaths a year in sub-Saharan Africa [7, 8]. An umbrella review conducted in Ethiopia revealed high burden of birth asphyxia (pooled prevalence 22.5%) in the country and has been noted the second leading cause of neonatal mortality next to prematurity [9]. Only 23% of asphyxiated babies have a chance of survival in developing countries [10].

Even though the majority of asphyxiated babies recovered, newborns who were exposed to prenatal hypoxia–ischemia for an extended period of time may suffer multi-organ damage [6]. Besides, prolonged hypoxia may lead to financial and emotional burdens on the families and communities [11] due to short term and long-term complications [4].

Perinatal asphyxia is multifactorial, including: Obstetric risk factors like: maternal age, antenatal care, maternal comorbidities, primiparity, having maternal fever, prolonged 2^nd^ stage of labor, and rupture of membrane, place of delivery [12–14]. Likewise neonatal related predictors for survival of asphyxia were sex, prematurity, low birth weight, delay presentation [15–17]. Depressed clinical status at admission, low 5^th^ min APGAR score, occurrences of infection and seizure within first day, hypothermia, hypoglycemia, hypoxemia, stage II and III of HIE, acute kidney injury, thrombocytopenia, [13, 18], were found to decrease the survival of asphyxiated babies significantly.

Even though various national programs have been undertaken to prevent birth asphyxia by providing high-quality prenatal, intra-natal, and postnatal care to every woman in the community, PNA remains a major problem in Ethiopia, with little data on recovery rates and predictors. Therefore, this study aimed to determine the survival status of asphyxiated neonates and potential predictors of time to recovery during the treatment periods of PNA in public hospitals of Addis Ababa, Ethiopia.

## Methods

### Study design, study area and study period

An institution-based retrospective follow-up study was conducted among asphyxiated newborns admitted in selected public hospitals in Addis Ababa. The study was carried out from February 15 to 15 March 2021 using records of admitted asphyxiated babies in NICUs from 1^st^ January 2016 to December 31^st^, 2020. The town has twelve government hospitals. Five hospitals belong to Addis Ababa Health Authority, four to the Federal Ministry of Health, one to the ministry of Education (AAU), and two to the defense force.

Of those eleven have a neonatal unit. The study was conducted in Tikur Anbesa Specialized hospital, Yekatit 12 hospital medical college, St. Peter Specialized Hospital and Gandhi memorial hospital which was selected by lottery method.

### Population

The source populations were all asphyxiated neonates who were admitted to the neonatal intensive care units of public hospitals of Addis Ababa and the study population were all randomly selected records of asphyxiated neonates admitted in the neonatal intensive care units of selected public hospitals of Addis Ababa from January 1, 2016, to December 31, 2020.

#### Inclusion and exclusion criteria

The study included all newborns with a confirmed diagnosis of asphyxia who were hospitalized to neonatal intensive care units of public hospitals in Addis Ababa between January 1, 2016, and December 31, 2020. And they were followed-up until they experienced the event or had reached the age of 28 days of postnatal. All asphyxiated neonates who had incomplete medical records and asphyxiated babies who had major congenital anomalies were excluded from this study.

### Sample size and sampling procedure

The sample size was determined using a double population proportion formula by considering gestational age, degree of asphyxia, birth weight and place of birth, as all these factors predict survival in newborns who are asphyxiated at birth [14]. Among those predictors, birth weight was found to be an independent predictor that gave a maximum of 395 sample size, by adding 10% for missing data gives a total sample size of 435.

Four public hospitals (Tikur-Anbesa specialized hospital, Yekatit 12 hospital medical college, Gandhi memorial hospital and St Peter specialized hospitals) were selected by lottery method from eleven public hospitals. The proportional allocation formula was used to select the study participants from each hospital and each year based on the five years total of the study period. After that, medical record numbers of babies diagnosed with perinatal asphyxia were identified from the registration logbook.

**Fig. 1:**
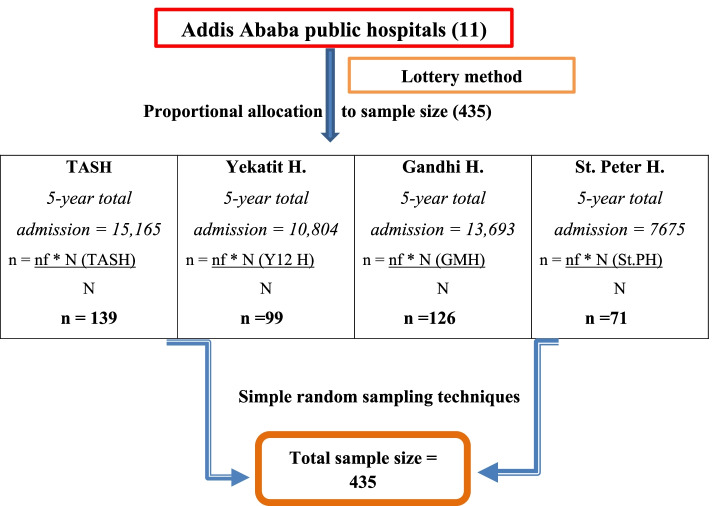
Schematic presentation of sampling procedure on survival status and predictors of survival in asphyxiated newborns admitted to NICUs of public hospitals, A.A, Ethiopia, 2021

Then, from the isolated medical record numbers in each hospital, computer-generated simple random sampling technique was applied to select the study participants (Fig. 1). Finally, the selected medical charts were reviewed from February 15 to March 15, 2021.

### Variables

Outcome variable is time to recovery dichotomized as (Recovered = 1 and censored = 0). Independent variables include, *Socio-demographic and Obstetrics factors*—maternal age, place of residency, gestational hypertension, antepartum hemorrhage, ANC follow up, parity, prolonged labor, prolonged rupture of membrane, mode of delivery and meconium stained amniotic fluid, *Neonatal related predictors*—Sex, gestational age, birth weight and age at clinical presentation, *Clinico–laboratory predictors*—Apgar score, crying, stage of HIE, clinical status at admission, infection or seizure occurrence within 24 h, vital-sign derangement, electrolyte disturbance and liver enzyme derangement, *Treatment-related predictors*—Bag mask ventilation, chest compression, Adrenalin, Aminophylline, fluid and electrolyte management.

### Operational definitions

*Perinatal asphyxia*—will be considered based on the clinical diagnosis made by a health care provider and recorded on the charts. *Stages of HIE*—it will be determined on the basis of Saranat’s classifications of the clinical diagnosis made by a health care provider (stage 1, stage 2 and stage 3). *Major congenital anomalies* – a wide range of abnormalities of body structure and function that present at birth, like heart defect, neural tube defect and Down’s syndrome. *Incidence density rate of survival* – It will be computed by dividing the number of events by total follow-up time in person-days. *Survival time* – number of days it takes from admission until a baby is recovered from PNA (that can be measured by using Incidence density rate and median recovery time). *Survival*—In this research, survival is newborns who become well (becoming free of clinical features of asphyxia, declared recovered from PNA and/or discharged from NICU due to completion of their management). *Censored*—are those newborns that had death summary sheet, against medical advice (Caregiver’s sign on behalf of their baby to leave the treatment before recovery), or referred to other health facilities. *Event*—newborns who recovered from PNA.

### Data collection tool and procedure

Structured data extraction format was adapted from the standardized HMIS registration book, PNA follow-up chart and other peer-reviewed articles [13, 14, 16, 19, 20]. Training was given to data collectors and supervisors regarding the significance of the study, truthful completion of the checklist and ethical considerations to standardize the data collection. Then; the records of all study participants were selected according to the eligibility criteria and all available information on patient records was checked. All relevant variables that meet the study objectives were extracted from patients’ charts by using a structured data extraction format. Treatment outcomes were confirmed by reviewing patient chart.

To ensure data quality, pretest was carried out with 5% (22 charts) of the study samples. Errors found during the verification process were corrected and modified before the analysis to ensure the agreement of the data abstraction format with the study objectives. All completed data collection forms were examined for completeness and consistency during data management, storage, cleaning and analysis. Consistency was also assessed by random selection of medical records by the principal investigator and cross-checking them for resemblance.

### Data processing and analysis

The collected data were coded, entered and cleaned in Epi data version 4.6 and then exported to STATA Version 16.0 statistical software for further analysis. Descriptive and inferential statistics were used to present the data. The necessary assumption of Cox-proportional hazard regression models was checked by using tests like Schoenfeld’s residual test. Kaplan Meier survival curve was used to estimate survival time, and the log-rank test was used to compare the survival curves among categorical predictors. Bi-variable Cox proportional hazards regression model was fitted for each explanatory variable. Then, those variables with a p-value ≤ 0.2 were fitted to multivariable Cox-regression analysis to identify independent predictors of survival of asphyxiated neonates. Hazard ratio with 95% confidence interval was used to measure the statistical significance association of predictors. In the multivariable Cox- regression analysis, variables having P-value < 0.05 were considered as significant predictors of survival of asphyxiated neonates. Finally, the results were summarized and presented in texts, tables and graphs.

## Result

### Socio-demographic & obstetric characteristics of mothers of study participants

Four hundred thirty-five charts of asphyxiated neonates were reviewed of which; 411(94.48%) were eligible in this study. Most of the mothers 351(85.4%) were between the age of 20 to 34 years with a median age of 26 years and a range of 16 to 40 years. One hundred fifteen (28%) of mothers gave birth via cesarean delivery (C-section). Antepartum hemorrhage, pregnancy induced hypertension, and history of abortion were the frequent Obstetrics & medical complications affecting (6%, 6.5%, 3.65%) of the pregnant women, respectively. Sixty-three (15%) of the babies had fetal distress during the intrapartum period and nearly half (46.7%) of the babies were born outside the admitting facilities (Table 1).

**Table 1:** Socio-demographic and Obstetrics characteristics of a pregnant mother who had admitted asphyxiated babies at NICUs of Addis Ababa public hospitals, (*n *= 411)

Covariates	Category	**Total **Number (%)	**Status**	
			**Survived **Number (%)	Censored Number (%)
Maternal Age	< 20 years	22(5.35)	18 (5.90)	4(3.77)
	20–34 years	351(85.40)	255(83.61)	96(90.57)
	> 34 years	38(9.25)	32(10.49)	6(5.66)
Place of residency	Addis Ababa	334(81.27)	247(80.98)	87(82.08)
	Out of Addis Ababa	77(18.73)	58(19.02)	19(17.92)
Parity	Primi-parous	239(58.15)	182(59.67)	57(53.77)
	Multiparous	172(41.85)	123(40.33)	49(46.23)
ANC follow up	No	10(2.43)	7(2.30)	3(2.83)
	Yes	401(97.57)	298(97.70)	103(97.17)
Place of delivery	Inborn	219(53.28)	153(50.16)	66(62.26)
	Out born	192(46.72)	152(49.84)	40(37.74)
Mode of delivery	SVD^a^	235(57.18)	172(56.39)	63(59.43)
	Assisted delivery	61(14.84)	46(15.08)	15(14.15)
	C/S delivery	115(27.98)	87(28.52)	28(26.42)
Duration of labor	Normal	354(86.13)	260(85.25)	94(88.68)
	Prolonged	57(13.87)	45(14.75)	12(11.32)
Duration of rupture of membrane	< 18 h	386(93.92)	286(93.77)	100(94.34)
	> 18 h	25(6.08)	19(6.23)	6(5.66)
Obstetrics & medical conditions	Cord problems	12(2.92)	8(2.62)	4(3.77)
	Hypertension	25(6.08)	15(4.92)	10(9.43)
	Pre/Eclampsia	22(88)	14(93.33)	8(80)
	APH^b^	15(3.65)	10(3.28)	5(4.72)
	Oligohydramnios	10(2.43)	6(1.97)	4(3.77)
	fetal distress	63(15.33)	55(18.03)	8(7.55)
	Multiple pregnancies	11(2.68)	7(2.30)	4(3.77)
	MSAF^c^	155(37.71)	122(40)	33(31.13)
	Abortion History	27(6.57)	16(5.25)	11(10.38)

^a^Spontaneous vaginal delivery, ^b^Antepartum hemorrhage, ^c^Meconium stained amniotic fluid

#### Neonatal characteristics of the study participants

Out of the cohort 411 (60.58%) were males. Most of the newborns (70%) had normal birth weight and 25.6% had low birth weight with a mean weight of 2.82 ± 0.65 kg. Nearly (15.3%) were preterm and 37(9%) were post-term. While comparing the mean age of neonates at a presentation on NICUs was prolonged in Out-born babies (9.6 ± 24.7) and censored (5.2 ± 16 h) compared to 0.9 ± 2.2 h for inborn babies and 4.9 ± 18 h for survived neonates from asphyxia. Around one-sixth (17%) of the babies cried at the time of birth and nearly 6% of neonates had a normal APGAR score (≥ 7) at the first minutes of life in both survivors and censored cases. Regarding the 5^th^ minutes, APGAR score, two-third (66%) of survivors and three-fourths (75%) of censored cases had low APGAR score (< 7) at the fifth minutes of life.

#### Clinical and laboratory characteristics of the study participants

Sixty-four and sixty-five presents of neonates presented in NICUs with altered consciousness and depressed Moro reflex, respectively. Forty-five percent of neonates admitted with a diagnosis of stage II HIE. From the total 99 (24.09%) newborns presented with Stage III HIE, only one out of eight of asphyxiated babies were survived and discharged alive.

Among censored neonates, 57.6% and 35.9% were admitted with Stage III and Stage II HIE, respectively. The most frequently identified additional medical complications at admission among asphyxiated newborns and during their hospital stays were hypothermia (86.13%), followed by respiratory distress (51.82%), MAS (64.96%), hypoglycemia (4.62%), sepsis (36.9%) and seizure disorders (23.1%).

The mean white blood cell count and hematocrit level were higher in censored babies than survived (21.74 ± 1.7 Vs 19.37 ± 0.58) and (55 ± 1.4 vs 53.74 ± 0.6), respectively. On the contrary, mean platelet count was lower in censored babies (171.62 ± 12 vs 196.76 ± 4.9) than survived babies. The mean serum sodium and calcium level in censored babies were lower than survived, but higher in Potassium, random sugar, AST, ALT and creatinine level within the first day of postnatal age (Table 2).

**Table 2:** Clinical and Laboratory characteristics of Asphyxiated babies who were admitted at NICUs of Addis Ababa public hospitals, Ethiopia 2021 [*n* = 411]

**Covariates**	**Category**	**Total **Number (%)	**Status**	
			**Survived **Number (%)	**Censored **Number (%)
Altered consciousness at admission		264(64.23)	172(56.39)	92(86.79)
Depressed Moro reflex at admission		270(65.69)	173(56.72)	97(91.51)
HIE stage at admission	Stage 1	127(30.9)	120(39.34)	7(6.60)
	Stage 2	185(45.01)	148(48.2)	38(35.85)
	Stage 3	99(24.09)	38(12.46)	61(57.55)
Additional medical diagnosis at admission	Respiratory distress	213(51.82)	156(51.15)	57(53.77)
	hypothermia	354(86.13)	255(83.61)	99(93.40)
	Hypoglycemia	19(4.62)	13(4.26)	6(5.66)
	MAS^a^	267(64.96)	195(63.93)	72(67.92)
Medical complication developed during their hospital stay	Hyperbilirubinemia	28(6.81)	26(8.52)	2(1.89)
	Necrotizing Enterocolitis	15(3.65)	9(2.95)	6(5.66)
	Acute kidney injury	23(5.6)	14(4.59)	9(8.49)
	Thrombocytopenia	20(4.87)	15(4.92)	5(4.72)
	Seizure	95(23.11)	71(23.28)	24(22.64)
	Sepsis (HAI)	152(36.98)	112(36.72)	40(37.74)
	Others^b^	19(4.62)	15(4.92)	4(3.77)
Laboratory investigation within 24 h of age (Mean ± SD)	WBC (cell/mm^3^) [*n* = 310]	19.81 ± 0.57	19.37 ± 0.58	21.74 ± 1.7
	HCT (%) [*n* = 310]	53.99 ± 0.57	53.74 ± 0.6	55 ± 1.4
	Platelet (10^3^) [*n* = 310]	191.96 ± 4.63	196.76 ± 4.9	171.62 ± 12
	Sodium (mEq/L) [*n* = 123]	136.5 ± 6.4	136.7 ± 6.3	135.2 ± 7
	Potassium(mEq/L) [116]	5.8 ± 1.4	5.8 ± 1.3	5.96 ± 1.7
	Calcium(mEq/L) [*n* = 60]	8.6 ± 2.1	8.8 ± 1.8	7.9 ± 3.1
	RBS (g/dl) [*n* = 241]	61.6 ± 67.8	61.4 ± 64.9	62.2 ± 75.5
	AST (U/L) [*n* = 57]	225.71 ± 41.4	214.2 ± 42.4	321.2 ± 165.5
	ALT (U/L) [*n* = 57]	73.82 ± 13.4	70.6 ± 12.8	100.5 ± 70.9
	Cr (mg/dl) [*n* = 186]	1.15 ± 0.06	1.12 ± 0.07	1.44 ± 0.12

^a^meconium aspiration syndrome, ^b^others (anemia, polycythemia, DIC and malnutrition)

### Treatment characteristics of the study participants

Majority of the newborns (88.32%) were resuscitated at birth, 59.12% were put on Oxygen and 7% were put on continuous positive airway pressure (CPAP) after resuscitation. About 112 (27.25%) neonates took anticonvulsant and 36 (8.76%) took aminophylline during their hospital stay. Two-third of the total fluid was prepared and administered to 65.7% of babies. One hundred twenty-nine (31.9%) babies received calcium gluconate with first days of life together with intravenous fluid.

### Overall proportion and incidence rate of survival of asphyxiated neonates

Of the total study participants, 305(74.2%) asphyxiated neonates were recovered or discharged alive from NICUs and 106(25.8%) were censored. Among censored neonates, 99(24.09%) died, 6(1.5%) left against and 1(0.24%) lost follow-up. The overall incidence density rate of survival of asphyxiated neonates was found to be 10 per 100 neonatal-days of observation (95% CI: 0.09–0.11).

### Time to recovery of neonates with asphyxia

**Fig. 2:**
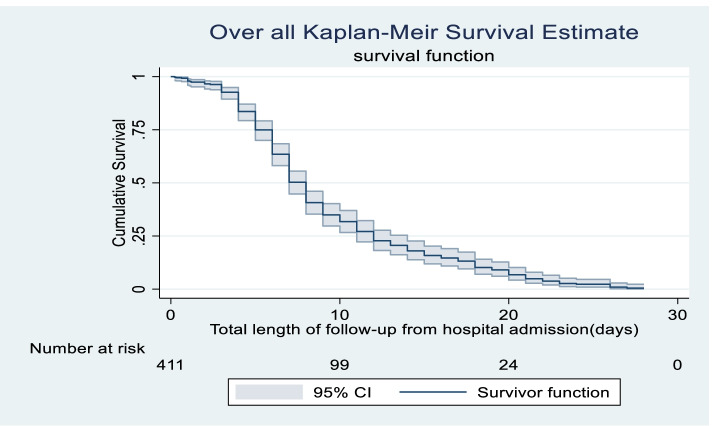
Overall Kaplan–Meier survival estimate of Asphyxiated neonates admitted in Addis Ababa public hospitals from 2016–2020, Addis Ababa, Ethiopia, 2021

Four hundred eleven admitted asphyxiated babies were followed for a total of 3062 neonate-days with a minimum of 1 h to a maximum of 28 days, with a median survival time of 8 days (95% CI: 7.527–8.473). The estimated cumulative probability of survival was 99%, 96%, 63%, 20%, 6% and 4% in 1,3,7,14,21 and 28 days, respectively (Fig. 2).

### Time to recovery among different groups of asphyxiated neonates

**Fig. 3:**
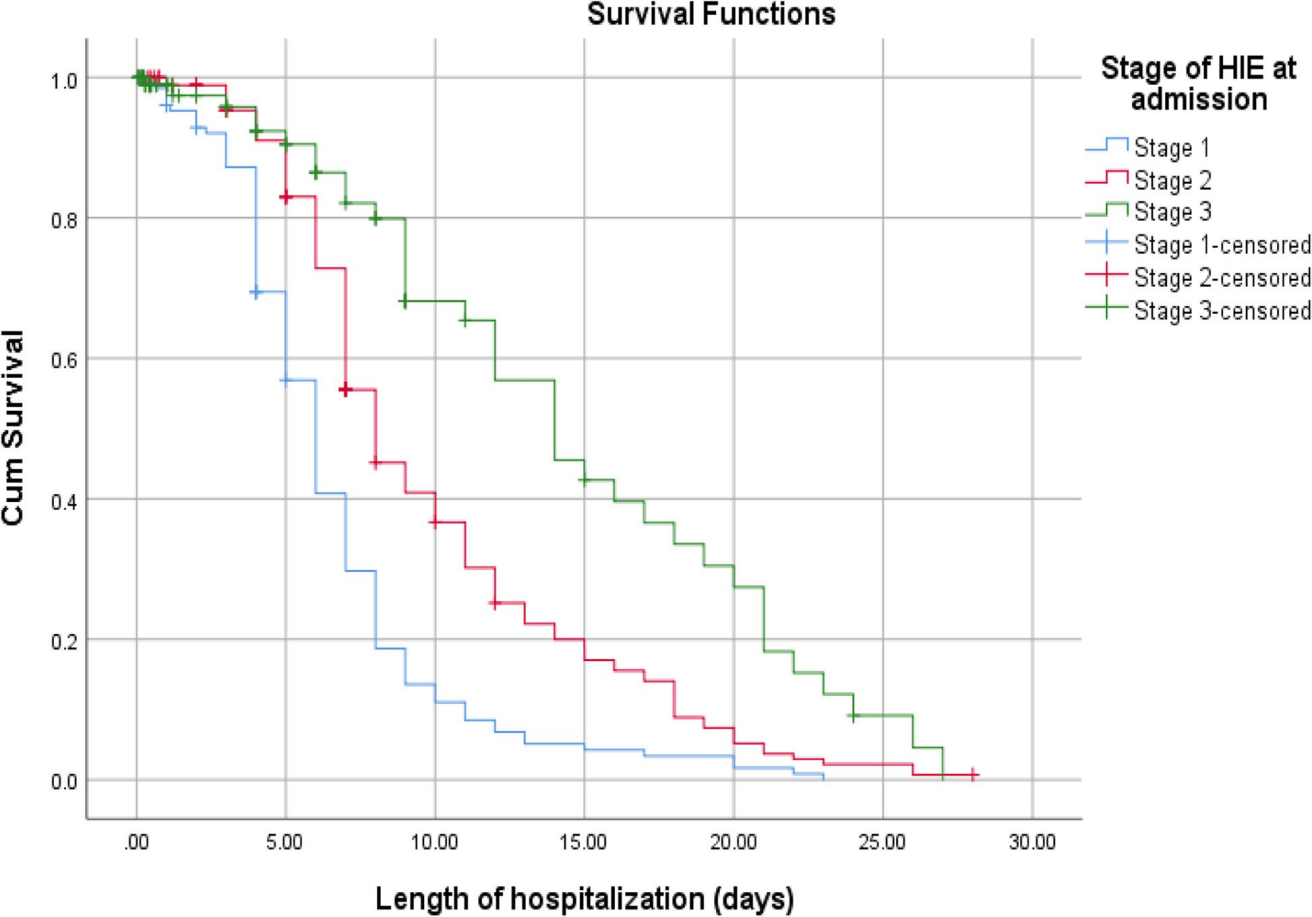
The KM survival curves comparing recovery time with different HIE stages among Asphyxiated neonates in Addis Ababa public hospitals, Ethiopia, 2016–2020

**Fig. 4:**
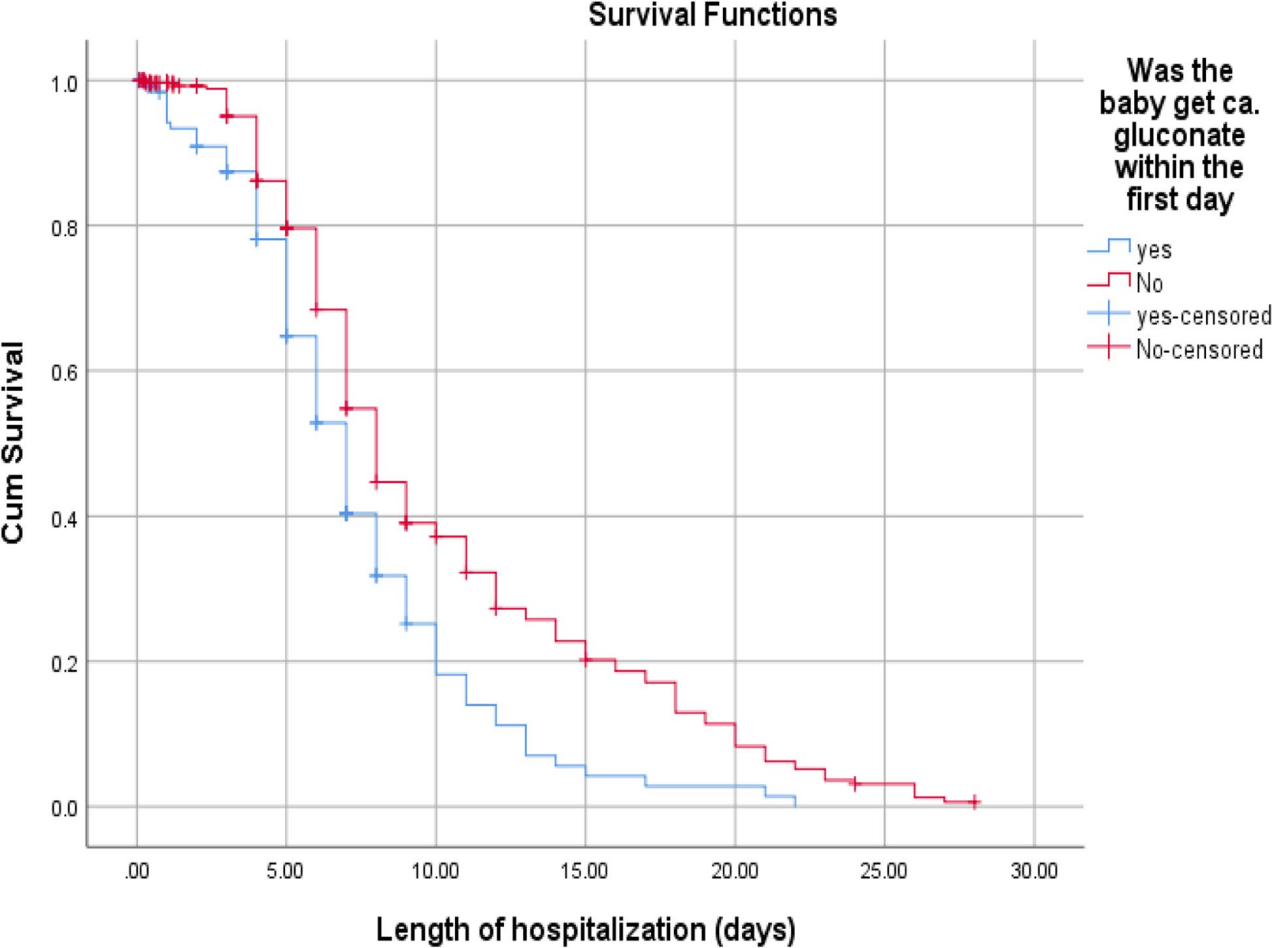
The KM survival curves comparing recovery time with different groups of Asphyxiated babies on ca +  + gluconate among neonates admitted to NICUs of Addis Ababa public hospitals, Ethiopia, 2016–2020

**Fig. 5:**
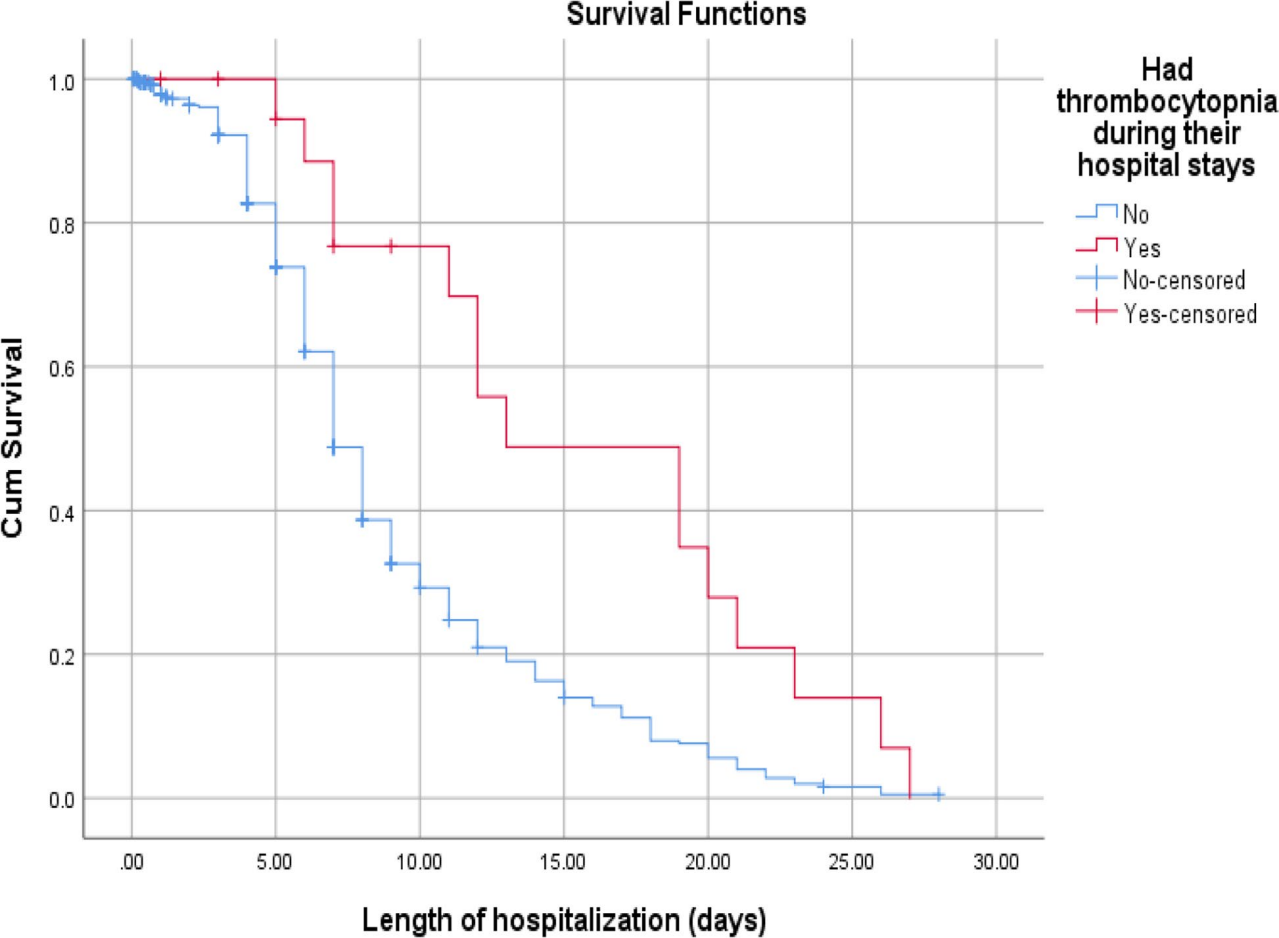
The KM survival curves comparing recovery time with different platelet counts among Asphyxiated neonates admitted in NICUs of Addis Ababa public hospitals, Ethiopia, 2016–2020

According to this study, the median recovery time for newborns diagnosed with stage 1 HIE were (6 days with 95% CI: 5.45–6.54) much faster than stage 2 and 3 HIE, (8 days 95% CI: 6.92–9.07) and (14 days 95% CI: 10.71–17.29), respectively. The median recovery time for low birth weight had longer than those of big and normal birth weight newborns (9 days Vs 6 and 7 days). Babies who got calcium gluconate within 24 h together with intravenous fluid slightly faster median recover time than counterparts (7 days 95% CI: 6.21–7.78) and (8 days 95% CI: 7.44–8.55), respectively. Furthermore, the median recovery time was nearly double in thrombocytopenic newborns than those who had a normal platelet count during their hospital stays (13 days Vs 7 days) (Figs. 3, 4 and 5).

### Predictors of survival status of asphyxiated neonates

In bi-variable Cox-regression, the socio-demographic and obstetric variables (maternal age, place of residency, mode of delivery and prolonged labor), Neonatal variables (birth weight, gestational age, age at presentation), clinico-laboratory variables (5^th^ minute APGAR score, HIE stages, Altered consciousness, depressed Moro reflex, sepsis, seizure, Meconium aspiration syndrome, hyperbilirubinemia, necrotizing enterocolitis, acute kidney injury and thrombocytopenia), treatment related variables (aminophylline, oxygenation, resuscitation and calcium gluconate) were found to be significant at p-value ≤ 0.2 and fitted for multivariable Cox-regression analysis.

Findings from multi-variable analysis showed that being low birth weight, stages of HIE, occurrence of seizure, thrombocytopenia and receiving calcium gluconate in the first day of life were identified predictors for recovery time from asphyxia.

Low birth weight neonates were found 33% less likely to recover faster from asphyxia compared to those with normal birth weight (AHR: 0.67, 95% CI: 0.47–0.96). The time of recovery was slower or prolonged when the stage of HIE stages increases. Neonates diagnosed with HIE stage II had 30% decrement and HIE stage III had 56% decrement in survival compared to Stage I HIE babies (AHR: 0.70, 95% CI: 0.51—0.97) and (AHR: 0.44, 95% CI: 0.27—0.71), respectively.

Regarding comorbidities, neonates who developed thrombocytopenia were 56% less likely to recover earlier than those of whom had a normal platelet count (AHR: 0.44, 95% CI: 0.24–0.80). Similarly, neonatal seizures that appear within 24 h of postnatal age were 39% and neonatal seizures that appear after 24 h of postnatal age were 31% lower probability to recover faster compared to neonates who had not experienced a seizure (AHR: 0.61, 95% CI: 0.38—0.97) and (AHR: 0.69, 95% CI: 0.49—0.97), respectively. Neonates who received calcium gluconate through intravenous fluid within 1^st^ days of life were hastened recovery time by 25% (AHR: 0.75, 95% CI: 0.58–0.99) (Table 3).

**Table 3:** Bivariate and Multivariate Cox regression analysis results of Asphyxiated babies who were admitted at NICUs of Addis Ababa public hospitals, Ethiopia, 2021 [*n* = 411]

Covariates	CHR (95% CI)	*P*-value	AHR (95% CI)	*P*- value
**Maternal Age**				
20 – 34 years	1		1	
< 20 years	0.94(0.58 – 1.52)	0.812	1.22(0.73—2.04)	0.455
> 34 years	1.34(0.92 – 1.94)	0.120	1.28(0.85—1.95)	0.241
**Place of residency**				
Addis Ababa	1		1	
Out of Addis Ababa	0.78(0.58 – 1.05)	0.105	0.89(0.64- 1.23)	0.479
**Mode of delivery**				
SVD	1		1	
Assisted delivery	0.92(0.66 – 1.27)	0.621	0.89(0.62—1.26)	0.506
C/S delivery	0.81(0.62 – 1.04)	0.107	0.89(0.66—1.19)	0.418
**Prolonged labor**				
No	1		1	
Yes	1.26(0.92 – 0.74)	0.142	1.21(0.85 -1.71)	0.292
**Birth weight**				
Normal	1		1	
Big baby	1.16( 0.71 – 1.91)	0.554	1.24(0.70—2.19)	0.454
Low birth weight	0.63(0.48—0.83)	0.001	0.67(0.47—0.96)	0.029 *
**Gestational Age**				
Term	1		1	
Post term	0.60( 0.42 – 0.85)	0.004	0.96(0.61—1.50)	0.841
Preterm	0.87(0.59 -1.29)	0.494	0.93(0.59—1.46)	0.753
**Age at presentation**				
< 24 h	1		1	
24 – 72 h	0.82(0.49 – 1.38)	0.470	0.45(0.16—1.23)	0.121
> 72 h	0.45(0.22—0.92)	0.031	0.64( 0.28—1.43)	0.275
**Fifth minutes APGAR**				
** ≥ 7**	1		1	
< 7	0.81(0.64—1.03)	0.091	1.03(0.78—1.35)	0.838
Meconium aspiration syndrome				
No	1		1	
Yes	0.83(0.65—1.05)	0.121	0.89(0.68—1.16)	0.386
**Hyperbilirubinemia**				
No	1		1	
Yes	0.76(0.51—1.14)	0.191	1.28(0.81—2.01)	0.285
**Necrotizing Enterocolitis**				
No	1		1	
Yes	0.46(0.24—0.91)	0.025	0.64(0.30—1.36)	0.245
**Acute kidney injury**				
No	1		1	
Yes	0.69(0.40—1.18)	0.171	0.90 (0.49—1.64)	0.726
**Thrombocytopenia**				
No	1		1	
Yes	0.45(0.26 -0.75)	0.003	0.44(0.24—0.80)	0.007 **
**Aminophylline**				
No	1		1	
Yes	0.48(0.26 -0.89)	0.019	0.80(0.40—1.58)	0.515
**Oxygenation**				
No	1		1	
Yes	0.57(0.40—0.82)	0.002	0.69(0.45—1.07)	0.095
**Resuscitation at delivery**				
No	1		1	
Yes	0.80(0.60—1.07	0.134	1.00(0.71—1.41)	0.994
**Ca**^**±±**^** gluconate**				
No	1		1	
Yes	0.61(0.48—0.79)	0.000	0.75(0.58—0.99)	0.039 *

*NB*:- *Significant (*P*-value < 0.05), **significant (*p*-value < 0.01) and *HR* = 1 is reference variable

Proportional hazard assumption was checked by using Schoenfeld’s residual global test. The findings indicated that all individual variables included in the model were satisfied PH assumptions (*p*-value > 0.05) and (Global test for Cox proportional hazard *P*-Value = 0.393 > 0.05).

### Discussion

Estimation of the median recovery time and identification of predictors of survival for asphyxiated newborns is crucial for the management and caring of asphyxiated neonates, especially for those resource-limited environments. From the total of 411 asphyxiated babies enrolled in this study 305(74.2%), neonates were survived and discharged from the NICUs. This finding is comparable with a cohort study conducted in Dire-Dawa public hospitals which were 74.3% [21].

On the other hand, this study's findings are lower than those of studies conducted in Cameron [22] and Ghana [23] in which 81.1% and 78.2% of neonates, respectively, recovered. Differences in the scope of the study, study sites, and study period could explain the disparity in results. Both trials took place in a single health facility over the course of a single year. Furthermore, the Cameron study was a case–control study that only employed the APGAR score to diagnose or identify the cases.

The overall median recovery time of NICUs admitted asphyxiated newborns in the cohort was found to be 8 days (95% CI: 7.527–8.473). This finding was consistent with the median time of recovery in studies performed in Dire-Dawa (7 days) and India (8 days), [21, 22]. However, it is longer than studies undertaken in Nigeria (3.8 days) [24] and Bangladesh (4.84 days) [25]. This discrepancy could be due to differences in health-care facilities, as the majority of their study participants were inborn babies, and the majority of the study participants were admitted to NICUs before the age of 12 h.

Several studies have confirmed low birth weight and preterm birth contributed to the poor prognosis of asphyxiated newborns (49–51). This study also revealed that low birth weight and preterm neonates have less chance of surviving compared to their counterparts. This could be explained by the fact that low birth weight babies are often preterm, and so lack sufficient surfactants, resulting in breathing difficulties, problems with cardiopulmonary transition, and consequent birth asphyxia. Furthermore, small babies have limited brown fat tissue, which raises their risk of hypothermia, which worsens hypoxia [5].

This result is lower than a study conducted in Nigeria, which was asphyxiated low birth weight babies were 2.2 times more likely to die [14]. This might be due to differences in sample size, study design and place of delivery. On the other hand, preterm babies have prolonged recovery time and a lower chance of surviving than term and post-term babies in our study but it has no statistical significance. This might be explained by differences in sample size, variable categorization, study area, and frequency percentage in prematurity (15.34% Vs 34.7%).

In the present study, neonates who were brought in the NICUs within 24 h recovered nearly three times faster compared to those who came after 24 h of their post-natal age. This finding is supported by studies conducted in Nigeria [16, 25], Nepal [6]. Unlike these findings, a study done in Pakistan showed that delayed presentation in NICUs was significantly associated with asphyxia morbidity and mortality [17]. This discrepancy might be due to a difference in the frequency of babies present within 24 h and the study population was only term babies.

Amongst the clinical and laboratory predictors, Stage of HIE, occurrence of seizure and thrombocytopenia were factors that influenced survivals and recovery time of asphyxiated babies. According to the findings of this study; recovery time and survival status of asphyxiated newborns are dependent on the stages of HIE. The incidence density rate of survival from stage I, stage II and stage III HIE were 14, 9 and 5.5 per 100 neonates- days’ observation, respectively. The chance of survival and faster recovery from asphyxia is higher in those neonates diagnosed with stage I HIE compared to stage II and stage III HIE.

This result was supported by other findings (15,33,35,42,43). This might be due to different reasons. Primarily, the central nervous system is one of the systems affected highly next to the renal system by asphyxia. When the stage of HIE increases; the primary and secondary energy failure happen which leads to the development of different neurologic sequelae and decrease the prognosis of the asphyxiated babies [26]**.** Secondly, the management is not most effective in most cases, once exposed to prolonged hypoxia and multiple organ damage occurs.

Lastly, neonates having an advanced stage of HIE mostly kept NPO (nothing per mouth) due to fear of necrotizing enterocolitis. This exacerbates malnutrition and thrombocytopenia, which may overcomplicate the existing problem with requiring additional time and resources to manage the complications related to this.

Other clinical related predictors that have a significant association with survival and recovery time from perinatal asphyxia are seizure occurrence. The occurrence of seizure in asphyxiated neonates usually associated with poor outcomes or indicates that the severity of encephalopathy is moderate or severe. This is supported by different literatures [18, 27, 28].

In this study*,* approximately 8.1% of asphyxiated newborns had seizure features within the first days of life and nearly 15.1% developed after 24 h of admission with a decrement of survival by 39% and 31%, respectively. However, a study conducted in Tanzania revealed that 28 percent of the newborn babies developed seizure within 24 h of age and 12.6% were beyond 24 h [18]. Despite the difference in study design, the difference may be explained by the difference in prevention mechanisms, like immediate and effective resuscitation in the labor ward, earlier management of metabolic derangement and appropriate usage of anticonvulsant.

According to the result of this study, thrombocytopenic-asphyxiated babies had a poor chance of survival than non-thrombocytopenic neonates. This may be due to an increase in the bleeding tendencies from the major organ, like the pulmonary, brain, and intestine. This is due to asphyxia impaired megakaryocytopoiesis and decreased platelet production, increased platelet consumption and sequestration [26]**.** This finding is supported by the clinical practice that most asphyxiated babies have moderate to severe thrombocytopenia and this cannot be easily managed with one or two platelet transfusions. Also, there is a scarcity of packs of platelets at any time when needed.

Lastly, the current study found that administration of calcium gluconate in the 1^st^ days of life together with intravenous fluid therapy has potentially beneficial effects for the baby and enhances the chance of survival than those who did not take prophylactic calcium gluconate. Hypocalcemia in newborns with asphyxia is mostly common for one of the following scientific reasons, low calcium intake, hyperphosphatemia, excess bicarbonate and functional hypoparathyroidism [1].

A randomized control trial was conducted to check the efficacy of prophylactic intravenous calcium administration in the first 5 days of life, it concludes symptoms of hypocalcemia and the requirement of hypocalcemia management were decreased in those newborns who took the intravenous calcium infusion [29].

The inclusion of multiple health institution and the ability to follow newborns from admission to discharge or death were the study's strengths. It was also expanded to include the previous five years. This can provide an opportunity to assess the existing situation and take any necessary remedial action to improve the survival of asphyxiated babies. It also takes into account clinico-laboratory predictors. This gives the researchers an idea to pursue future investigation. Beside the strength, this study was retrospective and depended on the infants' medical records. This could result in significant predictions being overlooked. Furthermore, it was carried out at public hospitals, which meant that newborns stayed at home and that private institutions were overlooked. Finally, excluding the incomplete medical records may also contribute to selection bias.

## Conclusion

In conclusion, the overall incidence rate of survival was 10 per 100 neonates-day observations with a median recovery time of 8 days. This indicates the recovery time was prolonged. Those neonates had low birth weight, stages of HIE, occurrence of seizure, thrombocytopenia and taking calcium gluconate in the first day of life were identified as a predictor for the survival time of asphyxiated newborns. Hence, early prevention, strict monitoring and taking appropriate measures timely is mandatory before babies transferred into highest stage of HIE and managing complications are recommended to hasten recovery time and increase survival of neonates.

## Supplementary Information

**Additional file 1.** 

**Additional file 2.** 

## Data Availability

The data sets used and/or analyzed during the current study are available as a supplementary file.

## Abbreviations

ALT: Alanine Aminotransferase
ANC: Antenatal Care
AOR: Adjusted Odds Ratio
APGAR: Appearance Pulse Rate Grimace Activity Respiration Rate
APH: Antepartum Hemorrhage
AST: Aspartate Aminotransferase
BMV: Bag-Mask Ventilation
CPAP: Continuous Positive Airway Pressure
HIE: Hypoxic Ischemic Encephalopathy
