# Supplementary material for: Survival and predictors of asphyxia among neonates admitted in neonatal intensive care units of public hospitals of Addis Ababa, Ethiopia, 2021: a retrospective follow-up study
Source: BMC Pediatr. 2022 May 10;22:262. doi: 10.1186/s12887-022-03238-w (PMC9087963; doi:10.1186/s12887-022-03238-w)
Supplement: Supplementary file 1 — Additional file 1. [file 12887_2022_3238_MOESM1_ESM.pdf]

APPENDIX

Annex I: Information sheet

This tool is prepared for the collection of socio-demographic, obstetric, neonatal, clinical, laboratory, treatment and outcome-related information that are important for the assessment of survival status and predictors of survival among asphyxiated neonates admitted in neonatal intensive care units of public hospitals, Addis Ababa, Ethiopia, 2021. This can provide empirical evidence for health professionals and program planners. And also, it can fill the information gap to some extent on survival and predictors of recovery from perinatal asphyxia. All this information was retrieved from the individual patient record without including the names of the clients and medical record numbers. This information was collected by health care providers (BSc Nurses) who are working at the hospital NICU clinics and the collected data will not be revealed to anyone except the principal investigators.

Lastly, if you want any more information, you can contact us at the address listed below.

Contact information:

1. Fekadeselassie Belege Cell phone +251912891577

Email address --- fekadebelege@gmail.com

Annex II: Data extraction form (Checklist)

Data collection date ____ /___/2013 E.c

Name of the Hospital --

Name of data collector ---------------------------------- signature------------------------------------

Name of supervisor -------------------------------signature---------------------------------------

Code no ---

Is baby asphyxiated **Yes** ☐ **No** ☐ **(If no stop filling the questioner)**

If yes, stage of PNA at initial diagnosis = ___________

Date of admission _____/____/_________ **E.c**

Admission diagnosis = ____

Part I: Socio demographic characteristic

**No**	**Socio demographic information**	**Possible answers**	**Remark**
101	Age of the mother	________ years	
102	Place of residency	1. Addis Ababa<br>2. Out of Addis Ababa	
	**PART-II Obstetric characteristics**		
201	Parity	________	
202	Gravida	________	
203	Previous history of abortion	1. Yes 2. No	
204	ANC visit	___________	
205	Place of delivery	1. Inborn 2. Out born	
206	Modes of delivery	1. Spontaneous Vertex delivery<br>2. Assisted Vaginal delivery<br>3. Caesarian section	
207	Duration of labor	______ hours ______ minutes	

208	Duration of ROM	1. ≤ 18 hours<br>2. > 18 hours		
209	Amniotic fluid	1. Clear<br>2. Meconium stained ____________ (grade)		
210	Type of pregnancy	1. Singleton<br>2. Twin<br>3. Other (specify)	______________	
211	Pregnancy related conditions	Anemia	1. Yes 2. No	
		Ante-partum hemorrhage	1. Yes 2. No	
		Gestational DM	1. Yes 2. No	
		Preeclampsia/ Eclampsia	1. Yes 2. No	
		Oligohydramnios	1. Yes 2. No	
		Fetal distress	1. Yes 2. No	
		Cord problems	1. Yes 2. No	
		If others, specify	____________________	
	**Part III: Neonatal characteristics**			
301	Sex of baby	1. Male 2. Female		
302	Gestational age	________ weeks ______ days		
303	Birth weight	____________ grams		
304	Did the baby cry	1. Yes 2. No		
306	Age at presentation	________ hours		
	**Part IV: Clinical and Laboratory characteristics**			
401	APGAR score	1. 1st min = __________ 2. 5th min = ___________<br>3. 10th min= ________ 4. 20th min =__________		
402	Depressed clinical status at admission	Level of consciousness	1. Normal<br>2. Lethargic<br>3. Hyper alert	
		Tone	1. Normal<br>2. Hypotonic	

			3. Hypertonic	
		Reflexes	1. Normal<br>2. Depressed<br>3. Exaggerated	
403	Vital sign, SpO_2 and RBS at admission, 6hr, 12hrs. and 24 hrs. after admission	1. Respiratory Rate	___, ___,<br>___, ___ b/min	
		2. Pulse rate	___, ___,<br>___, ___ b/min	
		3. Temperature rate	___, ___,<br>___, ___ ^{O}C	
		4. Oxygen saturation	___, ___,<br>___, ___ %	
		5. Random blood sugar	___, ___,<br>___, ___ mg/dl	
404	Serum electrolyte within 24 hours	Sodium	______ mmol/L	If get 2 result take the repeat one
		Potassium	______ mmol/L	
		Chloride	______ mmol/L	
		Calcium (Ionized)	______ mmol/L	
405	Complete blood count within 24 hours	White blood cell count	_____ k	
		Hematocrit	_____ %	
		Platelet	______ k	
406	Liver function test	Aspartate Aminotransferase (AST)	________ u/L	
		Alanine aminotransferase (ALT)	________ u/L	
		Total bilirubin	______ mg/dl	

407	Medical complications developed during their hospital stay	1. Hypoglycemia<br>2. Hyperbilirubinemia<br>3. Necrotizing enterocolitis<br>4. Acute kidney injury<br>5. Thrombocytopenia<br>6. Seizure<br>7. Sepsis (Hospital Acquired Infections)<br>8. Others ______________________________		
**Part V: Treatment related factors**				
501	How managed the baby	Resuscitation	1. Bag mask ventilation<br>2. Chest compression<br>3. Intubation<br>4. Adrenalin	
		Antibiotics	1. Yes<br>2. No	
		Aminophylline	1. Yes<br>2. No	
		Oxygen	1. Direct O_2<br>2. CPAP	
		Fluid management	1. Two third<br>2. Total fluid	
		Calcium gluconate with in first day of life	1. Yes<br>2. No	
**Part VI: Outcomes of the neonates and length of hospitalization**				
601	Final outcome	Recovered ☐ Censored ☐		
602	Date of discharge	____/_____/__________ E.c		
603	Length of hospitalization	___________ hours / days		
